# Fast Acoustic Light Sculpting for On‐Demand Maskless Lithography

**DOI:** 10.1002/advs.201900304

**Published:** 2019-05-15

**Authors:** Salvatore Surdo, Martí Duocastella

**Affiliations:** ^1^ Nanophysics Istituto Italiano di Tecnologia Via Morego 30 16163 Genova Italy

**Keywords:** acusto‐optics, direct‐write, laser‐interference, nanotechnology, optofluidics

## Abstract

Light interference is the primary enabler of a number of optical maskless techniques for the large‐scale processing of materials at the nanoscale. However, methods controlling interference phenomena can be limited in speed, ease of implementation, or the selection of pattern designs. Here, an optofluidic system that employs acoustic standing waves in a liquid to produce complex interference patterns at sub‐microsecond temporal resolution, faster than the pulse‐to‐pulse period of many commercial laser systems, is presented. By controlling the frequency of the acoustic waves and the motion of a translation stage, additive and subtractive direct‐writing of tailored patterns over cm^2^ areas with sub‐wavelength uniformity in periodicity and scalable spatial resolution, down to the nanometric range, are demonstrated. Such on‐the‐fly dynamic control of light enhances throughput and design flexibility of optical maskless lithography, helping to expand its application portfolio to areas as important as plasmonics, electronics, or metamaterials.

Optical techniques based on coherent interference of multiple beams allow to directly fabricate periodic sub‐wavelength structures over large areas with no need of masks or molds.^1^, ^2^, ^3^, ^4^, ^5^ Widely used strategies include interference lithography^3^, ^6^, ^7^ or direct laser interference patterning.^4^, ^8^ These techniques are capable of additive or subtractive nanofabrication while their throughput is threefold that of conventional direct‐laser writing methods. However, the Achilles heel of interference‐based processes is the speed of designing customized patterns. Typical implementations with diffractive optical elements,^9^ beam splitters,^4^, ^10^ Fresnel biprisms,^11^, ^12^ phase masks,^13^, ^14^ or mirrors^15^, ^16^ split light into a fixed number of beams at specific angles, resulting in static periodic patterns. Although mechanically displacing a single or multiple optical elements leads to changes in light interference, properly repositioning elements impedes real‐time selection of tailored designs. Alternatively, dynamic interference patterns can be generated with spatial light modulators (SLMs).^17^, ^18^ This enables individual phase selection for multiple beams, however, SLMs are polarization‐sensitive, suffer from pixelation, have low damage thresholds, and long response time (≈10 ms) preventing inter‐pulse pattern selection at repetition rates of commercial lasers.

To address the challenges encountered with existing techniques, we implemented an acousto‐optofluidics (AOF) system capable of generating periodic interference patterns at sub‐microsecond timescales. Combined with a fast translation stage, this enables on‐the‐fly direct‐writing of user‐selected sub‐wavelength structures with nanometer precision over indefinitely large areas, only limited by the stage travel range.

A scheme of our AOF system is shown in **Figure** **1**a. We assembled two pairs of parallel piezoelectric plates oriented along two orthogonal directions, namely *x*‐ and *y*‐axis, into a water‐filled cavity enclosed with optical windows transparent in the visible and infrared (IR) spectrum. By driving each piezoelectric pair with one or multiple sinusoidal signals—between 0.8 and 8 MHz in current experiments—vibrations are generated in the fluid, mainly along the *x* and *y* directions. At steady‐state, a standing acoustic wave is established in the cavity, and hence a periodic modulation in the density and refractive index of the liquid (Figure 1b), which behaves like a phase grating enabling dynamic generation of interference patterns.

**Figure 1:**
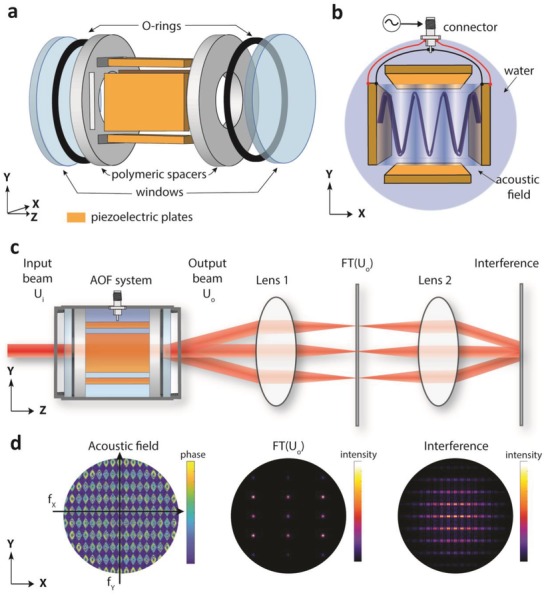
Working principle and experimental setup. a) Schematic of the main components of the acousto‐optofluidic system for light sculpting. For simplicity, the enclosed container and filling liquid are not included in the representation. b) Standing acoustic waves in the liquid generated by driving the *x*‐axis piezoelectric plates. c) Optical setup to generate interference patterns consisting of two converging lenses in a 4f configuration. The exit aperture of the AOF system is placed at the back focal plane of the first lens of the 4f system. d) Simulations of the phase or light intensity of a 635 nm Gaussian beam with a waist of 1 mm at 3 different locations of the optical setup: at the exit of the AOF system (acoustic field), at the Fourier plane of the first lens (FT(U_o_)), and at the Fourier plane of the second lens (interference).

The symmetry of the cavity determines the functional form of the acoustic waves in the fluid.^19^, ^20^ For a rectangular cavity, driving each piezoelectric pair with p different frequencies leads to a sinusoidally varying temporal and spatial refractive index that can be expressed as (see Figure S1, Supporting Information)

(1) $$n\;\left(x,y,t\right)=n_{0}\;+\sum_{i=1}^{p}n_{xi}\cos\left(\frac{\omega_{xi}}{c_{s}}x\right)\cos\left(\omega_{xi}t\right)+\sum_{i=1}^{p}n_{yi}\cos\left(\frac{\omega_{yi}}{c_{s}}y\right)\cos\left(\omega_{yi}t\right)$$

where *n*_0_ is the static refractive index, *c*_s_ is the speed of sound in the liquid, and ω_*xi*_, ω_*yi*_ are the resonant angular frequencies. The values of *n_xi_*, *n_yi_* correspond to the amplitude of the refractive index standing wave, and can be tuned with both amplitude and frequency of the driving signals. Specifically, they are maximized at the cavity resonance as in current experiments. Note that cavitation effects, namely the formation of bubbles when the fluid pressure decreases below its vapor pressure, limits the usable range of driving parameters (see Supporting Information). Figure 1c,d illustrates typical optical effects our AOF system induces on an incident coherent beam when implemented in a 4f‐system and modelled with scalar diffraction theory (see Supporting Information). In the Fourier plane of the first converging lens, the periodicity of Equation (1) results in splitting the beam into multiple beamlets (Raman–Nath diffraction), similarly to a tuneable diffraction grating. After the second converging lens, the beamlets interfere generating an intensity pattern.

We present various examples of experimental and simulated interference patterns for different wavelengths and driving conditions (**Figure** **2**; Figure S2, Supporting Information). Two main scenarios are possible based on the type of illumination. For laser pulses with duration shorter than the period of the driving signal, light interacts with the instantaneous refractive index. As a result, different patterns can be generated by simply changing the delay between laser pulses and acoustic standing wave (Figure 2a; Figure S3 and Video S1, Supporting Information). In agreement with simulations, when the change in refractive index is close to its maximum (Δ*t* = 50 ns), the interference pattern consists of hollow fringes (Figure 2b). It becomes progressively more sinusoidal with decreasing the change in refractive index, eventually disappearing when the standing wave is flat (Δ*t* = 160 ns). These results are due to the interference of multiple diffraction orders, with the weight of the highest orders decreasing with the refractive index variation. Interestingly, such multiorder interference causes light to be 3D structured (Figure 2c). A different scenario occurs for long laser pulses or continuous wave (CW) illumination. In this case, the time dependency of the refractive index cannot be resolved, and only time average patterns are observed. This obviates the need of synchronization and enables doubling the spatial density of lines in the patterns, but it comes with the caveat of reduced interference contrast. Still, adjusting the amplitude or frequency of the driving signals leads to interesting patterns of multiple arrayed features (Figure 2d).

**Figure 2:**
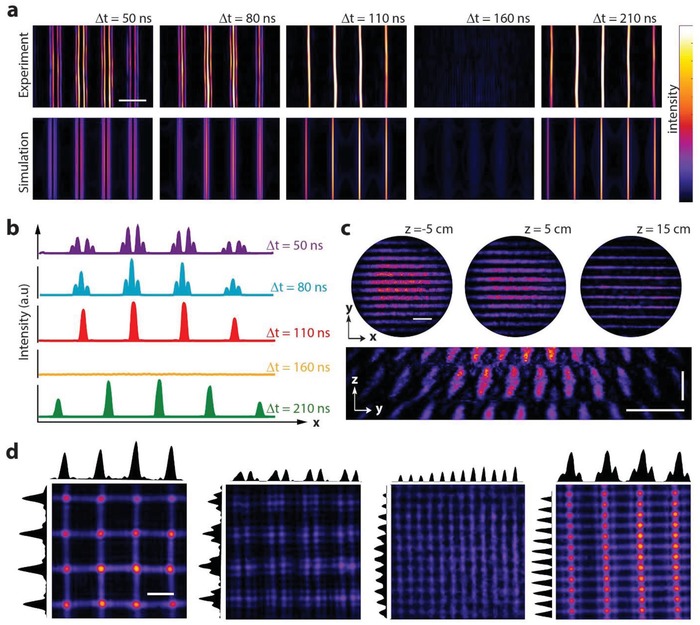
Light sculpting and modeling. a) Experimental images and simulations of different light patterns generated with synchronized pulsed illumination for an acoustic frequency of 1.5 MHz. Changing the time delay Δ*t* between laser pulse and the AOF system enables sub‐microsecond temporal interference control. b) Average intensity profiles from (a). c) 3D characterization of a light pattern generated at 4.5 MHz. Scale bar along *z*‐axis: 40 mm. d) Examples of interference patterns and averaged intensity profiles obtained with CW light at different driving frequencies. From left to right, in MHz: *f_X_* = 1.4, *f_y_* = 1.4; *f_X_* = 1.5, *f_y_* = 1.4; *f_X_* = 4.5, *f_y_* = 4.6; and *f_X_* = 1.4, *f_y_* = 4.5. All scale bars are 500 µm unless specified.

Critical parameters for AOF‐enabled laser patterning are optical wavelength range, stability, step response, and interference contrast (**Figure** **3**). Because the main limitation regarding wavelength range is the filling fluid absorption, it is possible to operate in the ultraviolet, visible, and IR by proper fluid selection. However, for a given acoustic frequency and low dispersive liquid, the pattern contrast slightly decreases with wavelength (Figure 3a). The step response, namely the time required to reach steady‐state, depends on the mechanical properties of the fluid and the quality of the cavity. In the current system, the step response is ≈600 µs (Figure 3b). This limits alternating interference patterns by rapidly changing the driving frequency to about 1.6 kHz. Nonetheless, once steady‐state is reached, synchronized pulsed light enables pattern selection at MHz rates (Figure 2a).

**Figure 3:**
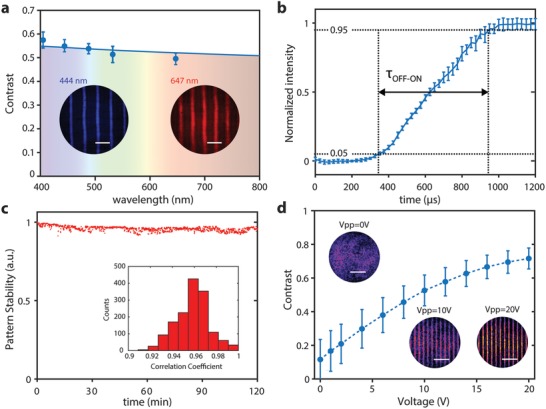
Optical characterization of the AOF system. a) Simulated (blue line) and measured (symbols) contrast of an interference pattern generated at the acoustic frequency of 1.5 MHz. Insets show interference generated with 444 and 647 nm CW lasers. b) Mean values (symbols) and standard deviation (bars) of the step response—from rest (OFF) to steady‐state (ON)—of the AOF system. c) Temporal stability of an interference pattern at the acoustic frequency of 1.2 MHz. The inset shows the histogram of the correlation coefficients used to calculate the pattern stability. d) Measured interference contrast for an acoustic frequency of 7.5 MHz and peak‐to‐peak voltage *V*_pp_ from 0 to 20 V. Insets show interferences at various voltages.

The high‐speed of the AOF system enables to rethink the traditional implementation of interference‐based patterning. Instead of irradiating an area with a fixed periodic pattern, we can now modify the interference pattern during exposure or, combined with a direct‐write station, change the pattern during stage scanning. **Figure** **4**a and Figure S4 (Supporting Information) show examples of ablated nanopatterns on palladium and silicon extending over an area of 0.5 × 1 cm^2^ (Figure S5a, Supporting Information). They were obtained by snake scanning the sample at 0.3 mm s^−1^ and alternating the acoustic frequency between 1.2 and 1.8 MHz. Regions (pixels) treated in this way show periodicities of 2.1 and 2.9 µm, respectively, in agreement with the acoustic frequency and magnification of the 4f‐system. In addition, ripples with feature sizes as small as 250 nm, less than one third of the 800 nm processing wavelength, are also present in all regions. They correspond to self‐organized structures caused by the interaction of incoming and reflected light at the surface, with a periodicity depending on the wavelength and angle of irradiation incidence.^21^, ^22^ Notably, the different pixels are seamlessly stitched, and display long‐range nanoscopic uniformity in the period (Figures S5 and S6, Supporting Information). Upon interaction with white light, the morphological differences between the patterns result in different colors, as shown in Figure 4b–d and Figure S7 (Supporting Information). Indeed, each pixel behaves as a reflective diffraction grating with a pitch dictated by the acoustic frequency (Figure S8, Supporting Information). The benefits offered by AOF for large‐area fabrication can be easily extended to 3D printing. Figure S9 of the Supporting Information shows examples of different photopolymerized structures covering a 1 × 1 mm^2^ area.

**Figure 4:**
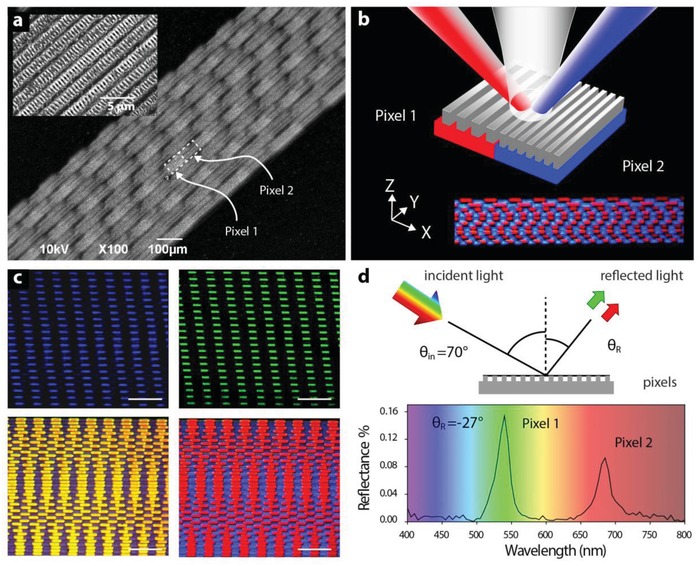
Large‐area structural coloring of metals. a) Scanning electron microscopy (SEM) image of a pattern with two distinct features (pixel 1 and pixel 2) obtained by snake scanning a palladium substrate while alternating the acoustic frequency between 1.2 and 1.8 MHz. The magnified SEM reveals nanoripples in the laser‐irradiated regions. b) Schematic of laser‐formed pixels consisting of periodic trenches with different spatial periods and hence structural colorations. The inset shows experimentally observed blue and red light simultaneously reflected by the array shown in (a). c) Examples of two large‐area (>4 mm^2^) patterns obtained as in (a) at different modulation rates (top, bottom). Two structural colors are observed by illuminating each pattern with a broadband light source at various angles of incidence. Scale bar 500 µm. d) Schematic of experimental setup used for reflectivity measurements (top). Experimental reflectance at θ_R_ = −27° of the array shown in (c) for an incident light with angle θ_in_ = 70°. Two distinct peaks in the spectrum confirm two structural colors on the same array (bottom).

The acoustic generation of light interference patterns at MHz rates enables combining, in a single setup, the rapid prototyping capabilities of direct‐writing with the large‐area processing and sub‐wavelength nature of interference‐based techniques. This overcomes the traditional trade‐off between spatial resolution, large‐area processing and design flexibility of optical maskless lithography systems. Acousto‐optic effects in birefringent crystals are typically employed for beam manipulation, but crystalline defects and frequency shifting of light complicate their use for interference generation.^23^ Our optofluidic system addresses these issues and offers ease of implementation and low cost. The linearity of the system leads to complex interference patterns by simple frequency addition, and the use of reverse engineering and off‐resonance operation should make possible the design of interesting light patterns.

We anticipate that the unprecedented speed of acousto‐optofluidic systems will lead to improvements in the throughput of interference‐based phenomena not only in maskless lithography, but also in several areas where the fast changing of light patterns plays a pivotal role. For instance, in superresolution microscopy techniques such as structured illumination microscopy^24^ or parallelized RESOLFT nanoscopy,^25^ the unique speed delivered by AOF systems could help maximize the amount of spatiotemporal information retrieved from a sample and, eventually, reduce photobleaching. In optogenetics, patterned photostimulation at sub‐ms time scales could help capture the fast dynamics of neuronal circuit activity and other relevant brain processes.^26^ Additionally, our system could help shape the future for the family of light‐activated chemical processes, ranging from to photoimmobilization^27^ to photoelectrochemistry,^28^ by offering an exceptional temporal control of light delivery.

## Experimental Section

*Assembly of the Acusto‐Optofluidic System*: Two pairs of parallel piezoelectric plates (APC International, 20 mm × 9.5 mm × 1.5 mm) were orthogonally held using 3D printed plastic supports (Figure S10, Supporting Information). The cavity was placed in a black metallic cylinder (SMS15, Thorlabs, diameter 2.54 cm) with appropriate electrical connections, and two optically transparent circular windows (WG41010, Thorlabs) were used to seal the device using O‐rings and epoxy (Hysol‐9483, Loctite). The cylinder shields the cavity from external electromagnetic waves while coaxial cables, which were used for delivering the driving signals to the piezoelectric plates, protect the AOF system from noise due to common‐impedance coupling. The cavity was filled with Mili‐Q water. Two arbitrary waveform generators with peak‐to‐peak dynamics of 20 V were used to drive the AOF system (GWInstek, AFG‐2112).

*Optical Characterization of the Acusto‐Optofluidic System*: A laser beam was expanded, collimated, and directed toward the AOF system. Interference patterns generated after a 4f‐system (f1 = 250 mm, f2 = 150 mm) were recorded using a CMOS camera (DCC1545M, Thorlabs) mounted on a translation stage. In synchronous mode, a 445 nm pulsed laser (CUBE 445‐40C, Coherent) was used to supply 5 ns long pulses synchronized with the driving signal of the AOF cavity. A digital pulse delay generator (SG‐535, Stanford Research Systems) was used to adjust the time delay between the driving signal and laser pulses. To record the average intensity patterns, the 445 nm pulsed laser operated in asynchronous mode and a 647 nm CW laser (OBIS LX, Coherent) were used as light sources.

Stability of the AOF system was measured by recording an image of an interference pattern every 5 s for 2 h, and calculating the correlation coefficient between the first and successive frames. The step response of the AOF system was characterized by recording interference patterns when driving the cavity with signals of different duration. The response time, τ_OFF − ON_, was calculated as the time required for the intensity of an interference pattern to rise from 5% to 95% of its steady‐state value. Contrast of the generated interference was evaluated as (*I*_max_ − *I*_min_)/(*I*_max_ + *I*_min_), with *I*_max_ and *I*_min_ being maximum and minimum intensity of the pattern.

*Laser Material Processing*: The laser direct‐writing (LDW) system for large‐area patterning consisted of a Ti:sapphire femtosecond laser (Coherent, 70 fs, λ = 800 nm), a microscope objective (Mitutotyo MPlan‐Apo, 50×/0.55NA), a homemade upright microscope, and the AOF system. The latter was conjugated to the sample plane by means of relay lenses. The sample was displaced relative to the interference pattern by a motorized *XYZ* stage with nanometer resolution (<10 nm), translation speed up to 6 mm s^−1^, and travel range of 11.2 × 11.2 × 0.73 cm. A CMOS camera (DCC1545M, Thorlabs) was coaxially coupled to the objective for real‐time sample inspection.

*Laser Ablation*: Periodic straight lines were ablated by moving the target substrate (palladium, silicon) with a respect to the interference pattern at a scan‐rate of 150 µm s^−1^. The repetition‐rate of the laser was 1 kHz and the beam‐energy was maintained between 4 and 4.4 µJ with a rotating λ/2‐wave plate and a polarizing beam splitter.

*Two‐Photon Lithography*: Pentaerythritol triacrylate (PETA, Sigma‐Aldrich) was used as negative resin for polymerization and isopropylthioxanthone (ITX, Sigma‐Aldrich) as photoinitiator. The photoresist composition was 0.08% of ITX in PETA. In lithography experiments, a drop of photoresist was squeezed between two glass‐cover slips mounted on the stage of the LDW system. After irradiation with the interference pattern (energy per pulse of 0.3 µJ), the sample was immersed in methanol for 5 min and rinsed with isopropanol to wash away the liquid monomer.

*Samples Analysis*: Morphology of laser‐irradiated samples was investigated with scanning electron microscopy (JSM‐6390, JEOL) at an acceleration voltage of 10 kV. Polymeric samples were sputter‐coated with 10 nm of gold in order to inhibit charging effects. 3D images of polymeric microstructures were collected with an optical profilometer (Zeta‐20, Zeta Instruments). AFM images were acquired in air with an atomic force microscope (MFP‐3D, Asylum Research) in tapping mode. The probes used (PPP‐NCHR, Nanosensors) were aluminum‐coated silicon cantilevers with nominal resonance frequency of 330 kHz, spring constant of 40 N m^−1^, and tip‐radius 5 nm. The images (amplitude data) were collected by scanning 20 × 20 and 6 × 6 µm^2^ areas with a resolution of 256 × 256 pixel.

Characterization of laser‐induced structural colorations was carried out by means of scatterometry. Pixel reflectivity for various scatter angles and excitation wavelengths was acquired with a photospectrometer (V‐VASE, Woollam) at angular and spectral resolution of 0.1° and 5 nm. The angle of incidence of light was 70° with respect to the normal of the specimen. Pixel reflectivity was measured for scatter angles from −40° to −10° and for incident wavelengths of 432, 532, and 633 nm. Reflectivity spectra in the range of 400–800 nm were collected at a scatter angle of −27° and normalized to the intensity of the incident light.

## Conflict of Interest

The authors declare no conflict of interest.

## Supporting information

Supplementary
