# Supplementary material for: Fast Acoustic Light Sculpting for On‐Demand Maskless Lithography
Source: Adv Sci (Weinh). 2019 May 15;6(14):1900304. doi: 10.1002/advs.201900304 (PMC6662050; doi:10.1002/advs.201900304)
Supplement: Supplementary file 1 — Supplementary [file ADVS-6-1900304-s001.pdf]

Copyright WILEY-VCH Verlag GmbH & Co. KGaA, 69469 Weinheim, Germany, 2019.

ADVANCED SCIENCE
Open Access

Supporting Information

for *Adv. Sci.,* DOI: 10.1002/advs.201900304

Fast Acoustic Light Sculpting for On-Demand Maskless Lithography

*Salvatore Surdo and Martí Duocastella**

Supplementary information:

Fast Acoustic Light Sculpting for On-demand Maskless Lithography

Salvatore Surdo, Martí Duocastella

Nanophysics, Istituto Italiano di Tecnologia, Via Morego 30, 16163 Genoa, Italy

1 Acousto-optofluidics for interference generation

In this section, we provide a theoretical foundation for generating interfererence patterns using acoustic waves in a liquid. The section is divided into three parts. First, we use fluid mechanics to describe the spatial and temporal changes in refractive index induced in a liquid. Second, by using scalar diffraction theory, we explain how such refractive index variations affect light propagation. Finally, we discuss some of the properties required for the filling fluid, with special emphasis in how to prevent cavitation.

1.1 Acoustic generation of refractive index gradients in a liquid

For simplicity, we will first derive spatially and temporally changes in refractive index induced when driving a pair of piezoelectric plates along the x-axis with a single frequency. By neglecting cross-talk with the y-axis and considering the superposition principle, we will then get to a generalized solution corresponding to driving both x and y plates with p different frequencies.

Considering that acoustic waves induce small changes in the density of the liquid, the linearized damped wave equation can be written as [1]:

$$\nabla^2(c_s^2\rho + \nu\frac{\partial\rho}{\partial t}) - \frac{\partial^2\rho}{\partial t^2} = 0 \tag{1}$$

where ρ is the change in density, c_s the speed of sound and ν the effective kinematic viscosity of the liquid defined as: $\nu = (4/3\mu + \mu_b)/\rho_0$, with μ being the dynamic shear visocity, μ_b the dynamic bulk viscosity, and ρ_0 the static density of the liquid. The corresponding one dimensional equation (x-axis) in Cartesian coordinates is:

$$c_s^2\left(\frac{\partial^2\rho}{\partial x^2}\right) + \nu\left(\frac{\partial^2(\frac{\partial\rho}{\partial t})}{\partial x^2}\right) - \frac{\partial^2\rho}{\partial t^2} = 0 \tag{2}$$

We can write the continuous input of energy provided by a driving signal of angular

frequency ω_x as a non-homogeneous Neumann boundary condition [1, 2]:

$$\left.\frac{\partial \rho}{\partial x}\right|_{x=0;x=L} = \frac{\rho_0 v_A \omega_x}{\nu^2 \omega_x^2 + c_s^4}\left[c^2 sin(\omega_x t) - \omega_x \nu cos(\omega_x t)\right] \tag{3}$$

where v_A is the amplitude of the inner wall velocity of the piezoelectric plates located at positions 0 and L, which is in the order of 10 μm/s for voltage amplitudes of 10 V, similarly to what was applied in our experiments (Figure S1a). The corresponding initial conditions of this system can be described by two functions:

$$\rho(x,0) = g(x) \tag{4}$$

$$\left.\frac{\partial \rho(x,t)}{\partial t}\right|_{t=0} = h(x) \tag{5}$$

where $g(x) = h(x) = 0$ when switching ON the AOF system. The solution of this partial differential equation (PDE) with non homogenous boundary conditions can be found as the sum of an auxiliary function and the solution of a PDE with homogeneous boundary conditions. Using the Storm-Liouville theory leads to:

$$\begin{gathered}\rho(x,t) = x(Acos(\omega_x t) + Bsin(\omega_x t)) + \\ \sum_{n=1}^{\infty} cos(k_n x)\left\{e^{-\nu k_n^2 t/2}\left[C_n cos\left(\frac{t k_n^2}{2}\sqrt{4c_s^2 - \nu^2 k_n^4)}\right) + \right.\right. \\ \left.\left. D_n sin\left(\frac{t k_n^2}{2}\sqrt{4c_s^2 - \nu^2 k_n^4}\right)\right] + E_n cos(\omega_x t) + G_n sin(\omega_x t)\right\}\end{gathered} \tag{6}$$

where $k_n = \frac{n\pi}{L}$ correspond to the eigenvalues, C_n and D_n are determined by the initial conditions, A and B from the boundary conditions and E_n and G_n from substituing into equation 2. As expected, the exponential term corresponds to Stoke's law of sound attenuation, and thus its contribution at steady-state can be neglected. When driving the piezoelectrics at the m-th resonant frequency ($\omega_x = c_s k_m$, with m being an odd number), the low viscosity regime can be considered, and the only term contributing to the final solution is E_n. In this case, the steady-state density fluctutations correspond to a standing wave (Figure S1b) given by:

$$\rho(x,t) = \frac{4}{\nu}\frac{L v_A \rho_0}{m^2 \pi^2} cos(k_m x) cos(\omega_x t) \tag{7}$$

Considering the Lorentz-Lorenz equation, the relationship between density and refrac-

tive index can be written as:

$$n = n_0 + \frac{n_0^4 + n_0^2 - 2}{6n_0}\left(\frac{\rho}{\rho_0}\right) \tag{8}$$

Therefore, the spatiotemporal change in density from equation 7 can be expressed as a periodic change in refractive index:

$$n(x,t) = n_0 + n_A cos(\frac{\omega_x}{c}x)cos(\omega_x t) \tag{9}$$

where:

$$n_A = \frac{2}{3}\frac{v_A c_s^2}{\nu\omega^2 L}\frac{n_0^4 + n_0^2 - 2}{n_0} \tag{10}$$

For water, piezoelectric plates separated by 1 cm and driven at 1 MHz and a voltage amplitude of 10 V would lead to a local change in the refractive index with amplitude $n_A \approx 2.10^{-5}$. Importantly, the amplitude of the driving signal determines the value of v_A, and thus the change in refractive index can be electronically controlled, which forms the basis of our approach for the tunable generation of interference patterns.

Applying the principle of superposition and neglecting any crosstalk between the piezoelectric pairs along the x- and y-axis, leads to the generalized equation 9 for the case of p different resonant frequencies along each axis:

$$\boxed{n(x,y,t) = n_0 + \sum_{i=1}^{p} n_{Axi} cos(\frac{\omega_{xi}}{c_s}x)cos(\omega_{xi}t) + \sum_{i=1}^{p} n_{Ayi} cos(\frac{\omega_{yi}}{c_s}x)cos(\omega_{yi}t)}$$

where n_{Axi} and n_{Ayi} correspond to amplitude changes in refractive index for each frequency. Notably, each of these values can be independently controlled by adjusting the respective driving signals.

1.2 Light diffraction theory

The wavelength of the acoustic waves induced in the cavity is large compared to optical wavelengths. Thus, the optical response of our AOF system can be properly modeled using scalar diffraction theory [3]. In particular, by neglecting the absorption of light from the liquid, light passing through the AOF system experiences a phase transformation:

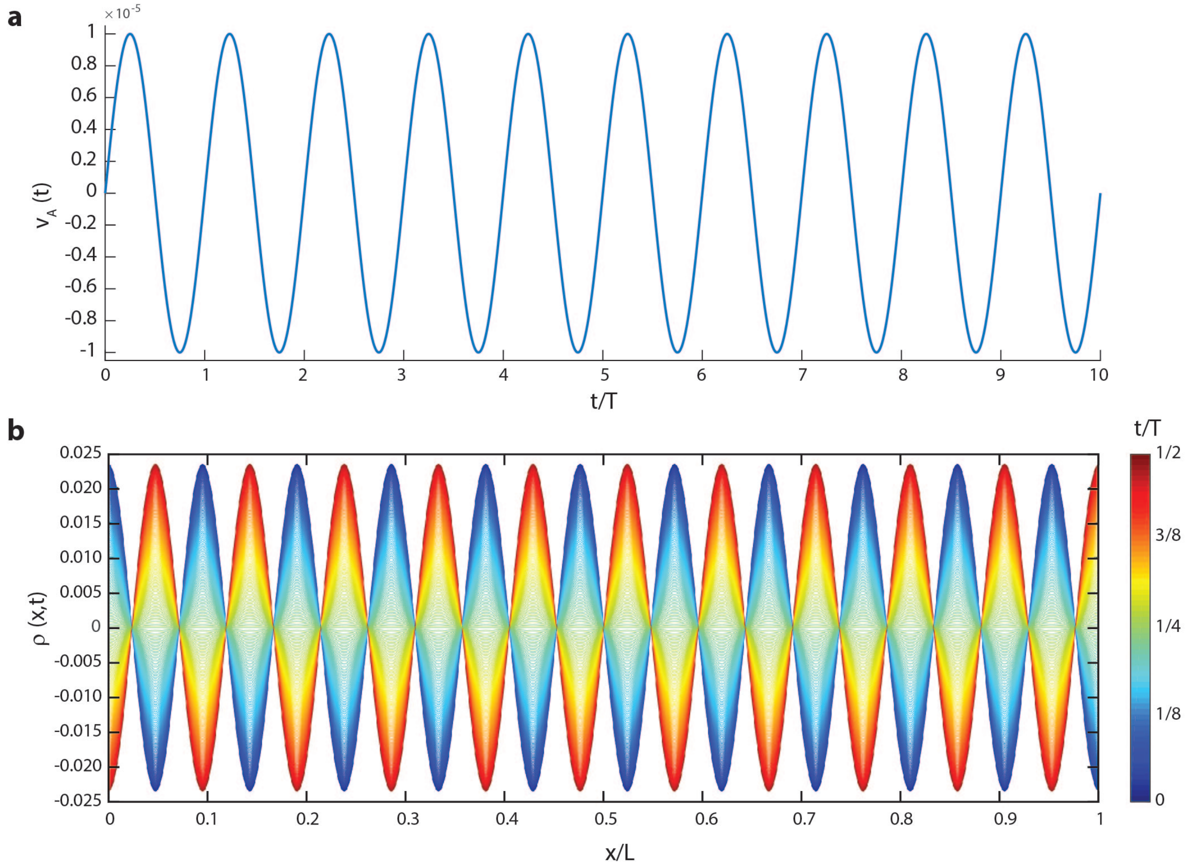

Figure S1. Acoustic generation in the AOF system. a) Simulated oscillogram of the piezoelectric wall velocity (in m/s) versus normalized time (time over period of oscillation T) when driven at a resonance frequency of 1.5 MHz. b) Simulated density standing waves (in kg/m^3) generated inside the cavity when driven with the signal corresponding to (a). The colorbar indicates different time points.The position of the standing waves (x) has been normalized with respect to the cavity spacing L.

$$\theta(x, y, t) = kln(x, y, t) \tag{11}$$

where k is the wavenumber of the light and l the length of the cavity. Because of the limited physical aperture of the AOF system, the complex electric field U_o of a monochromatic plane wave after passing through it is given by [4]:

$$U_o(x, y, t) = U_i(x, y)P(x, y)e^{ikln(x,y,t)} \text{,with } P(x, y) = \begin{cases} 1, & for\ \sqrt{x^2 + y^2} \leq D \\ 0, & for\ \sqrt{x^2 + y^2} > D \end{cases} \tag{12}$$

where U_i is the incident electric field and D the diameter of the aperture of the system. By using the Fresnel diffraction integral, the field U_f at any arbitrary distance z from the AOF

device can be calculated as:

$$U_f(u,v,z,t) = \frac{e^{ikz}}{i\lambda z}\int_{\infty}^{-\infty}\int_{\infty}^{-\infty} U_o(x,y)e^{\frac{ik}{2z}[(u-x)^2-(v-y)^2]}dxdy \tag{13}$$

where the quadratic phase term corresponds to the Fresnel kernel. In case of using lenses to focus the light at the output of the AOF system, equation 13 still applies, provided the addition of a phase term to account for the lens response. Interestingly, at the focal plane of the first lens that integrates the 4f system used in current experiments, the Fresnel integral can be reduced to a simple Fourier transform:

$$U_f(u,v,z,t) = \frac{1}{\lambda f}\int_{\infty}^{-\infty}\int_{\infty}^{-\infty} U_o(x,y)e^{\frac{ik}{f}[xu+yv]}dxdy \tag{14}$$

where f_1 is the focal length and the spatial frequencies u and v are related to the coordinates of the input spectrum at $\frac{u}{\lambda f}$ and $\frac{v}{\lambda f}$. At the focal plane of the second lens of the 4f system, the Fourier relationship from Equation 14 also holds. Given the Fourier transform properties, at this position we have an image of the acoustic field described by Equation 12, with coordinates (x', y') given by $(x/M, y/M)$, where $M = \frac{f_1}{f_2}$ corresponds to the magnification of the 4f system.

1.3 Filling-fluid selection considerations: cavitation issues

Besides the static refractive index, density, and optical transmittance, another key parameter to consider for the filling fluid is its vapor pressure. Indeed, pressure gradients induced within the cavity can lead to cavitation, namely the formation of bubbles when the liquid pressure drops below its vapor pressure. In general, the dimensionless parameter CA (cavitation number) is used to quantify the probability of cavitation events:

$$CA = \frac{P_i - P_v}{\frac{1}{2}\rho(x,t)c_s^2} \tag{15}$$

where, P_i and P_v are the initial pressure within the cavity and liquid vapor pressure, respectively. In general, cavitation occurs for $CA << 1$. By combining Equation 7 with Equation 15, we can determine the maximum probability of cavitation based on the paramters of the filling fluid and driving signal:

$$CA = \frac{1}{2}\frac{\nu\omega^2 L\rho_0}{v_A c_s^4}(P_i - P_v) \tag{16}$$

At conditions used in current manuscript ($P_i \sim 101kPa$, $P_v \sim 2.8kPa$), $CA > 1$ and cavitation effects can be excluded, in agreement with experimental observations. Note, though, that cavitation probablity increases with the driving signal (incerase in v_A) and by operating at lower frequencies. In any case, the risk of cavitation can be averted by selecting a filling fluid with lower vapor pressure or by pressurizing the cavity (increasing P_i).

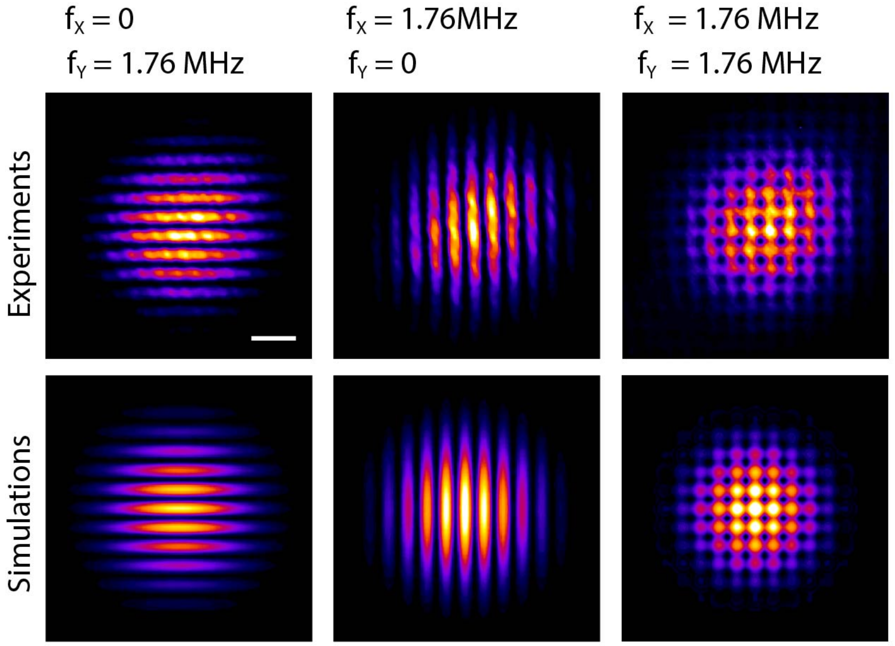

Figure S2. Interference pattern generation with a CW laser. Experimentally observed (top) and simulated (bottom) interference patterns generated by using an acoustically shaped 647-nm CW laser and a 4f-imaging system (f1=250 mm , f2=150 mm). Scale bar 200 μm.

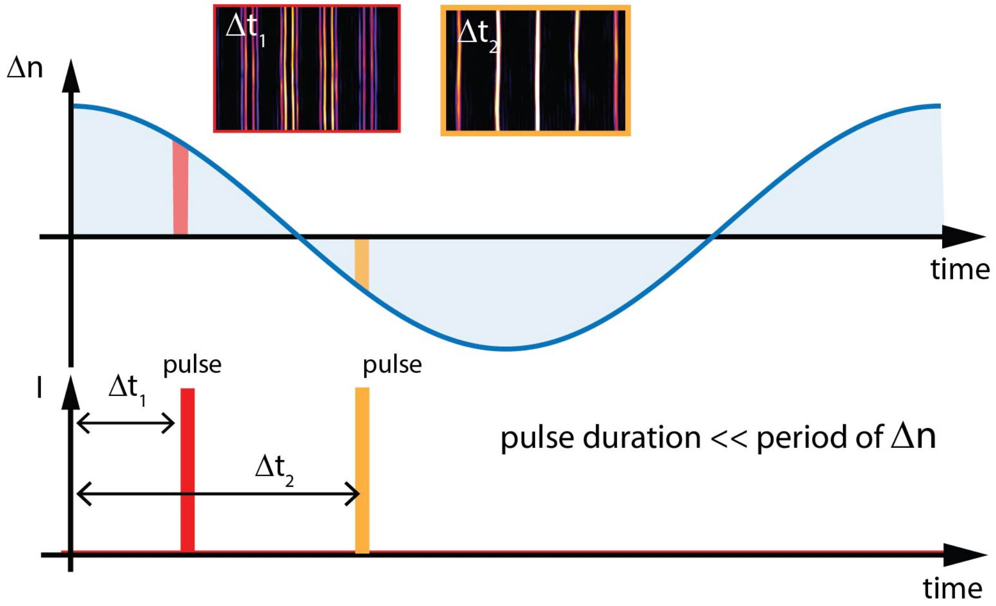

Figure S3. Signals for interference generation using synchronized pulsed light. By changing the time delay between the periodic change in refractive index and laser pulses enables selecting different interference patterns with sub-microsecond temporal resolution.

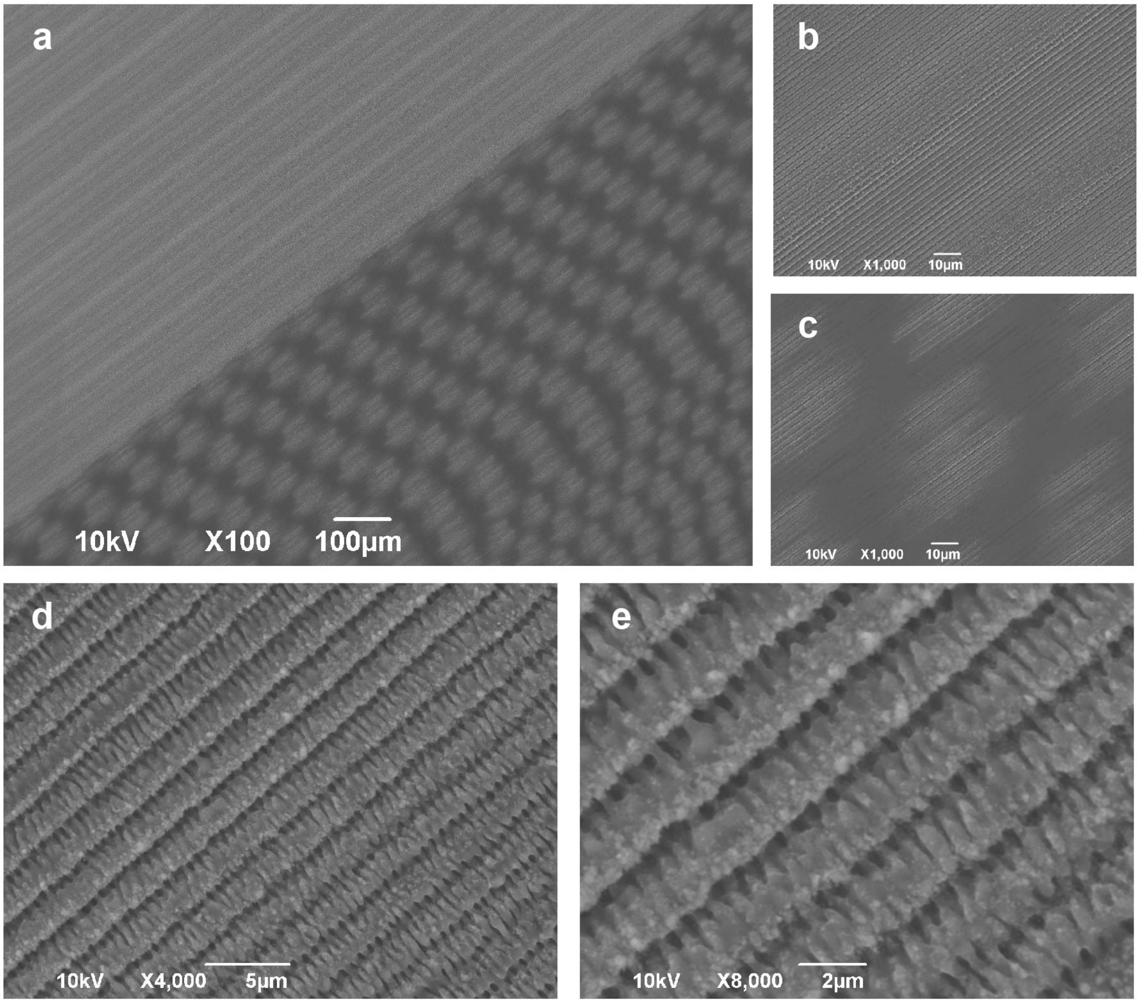

Figure S4. Large-area laser nanopatterning of silicon. a) Scanning electron micrograph of a continuous (top) and binary modulated (bottom) patterns obtained by snake scanning at 0.3 mm/s an 800-nm pulsed laser acoustically shaped at 1.2 MHz. Details of continuous pattern (b) and modulated pattern (c). d,e) Scanning electron microscopy at high magnification reveals nanometric features on the laser-irradiated regions.

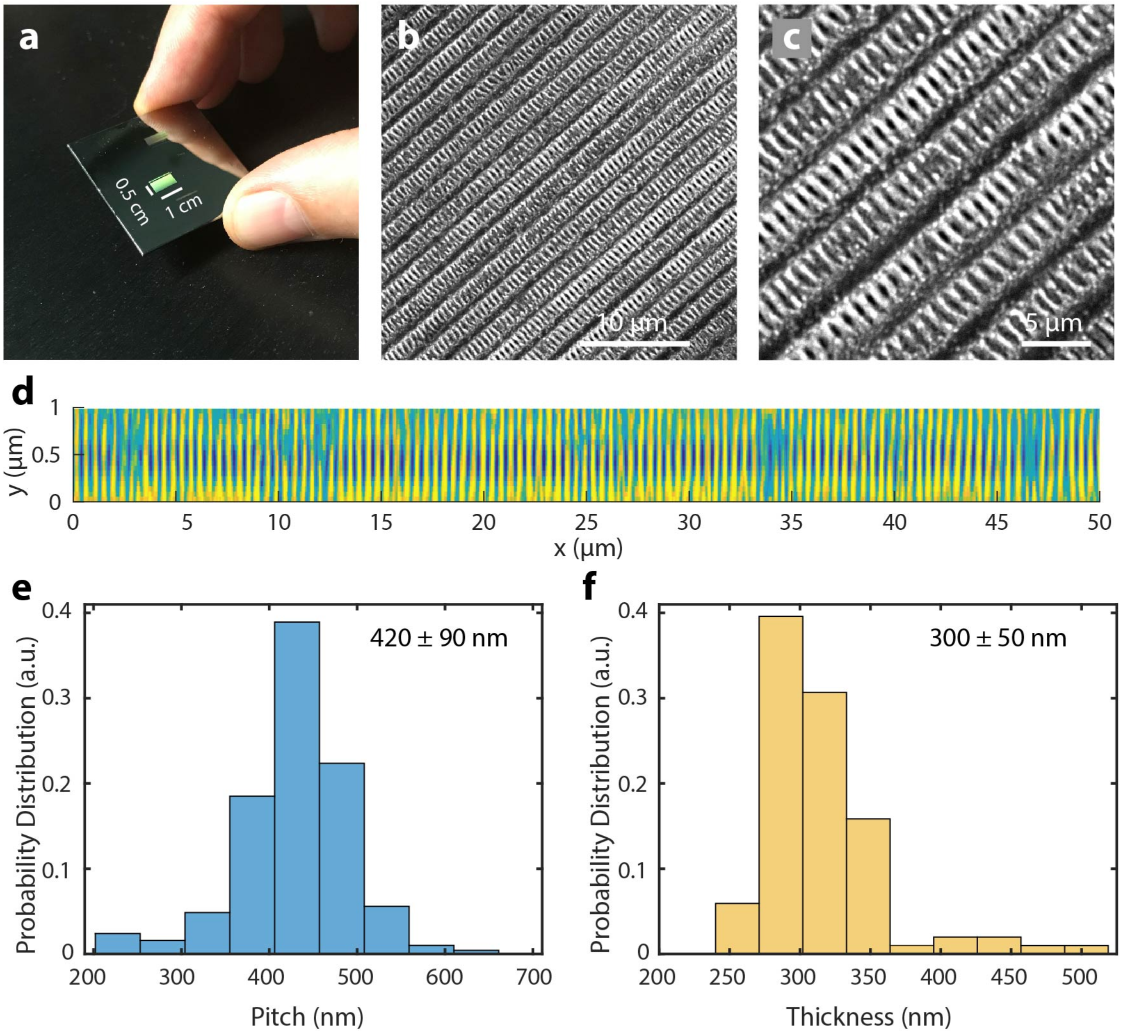

Figure S5. Morphological characterization of laser-formed nanopatterns. a) Optical image of a large (0.5 $\times 1\text{cm}^2$) structural coloration onto a palladium substrate. b,c) Scanning electron micrographs of a portion of the structural colored region shown in (a) and revealing nanoripples on the laser-irradiated area. d) Intensity profile of several (>100) nanoripples within an individual fringe of the interference pattern. Distribution of pitch (e) and width (f) of the laser-generated nanoripples shown in (d).

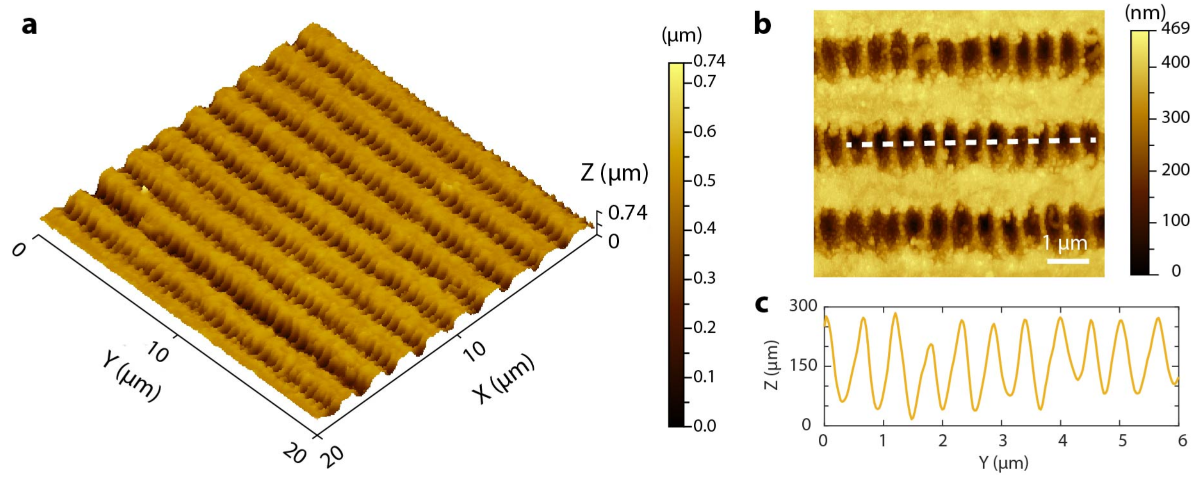

Figure S6. Atomic force microscopy (AFM) characterization of the laser-formed palladium nanopatterns. a) Topography map of patterns containing nanoripples obtained with an acoustic frequency of 1.2 MHz. b) Detail of the structure shown in a) obtained with higher resolution. c) Cross-section profile corresponding to the dashed white line in b).

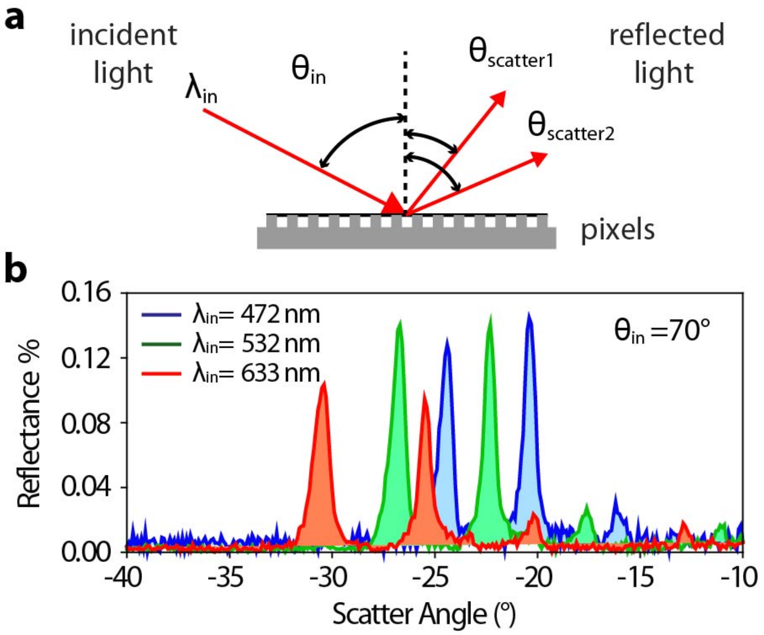

Figure S7. Quantificaiton of laser-induced structural coloration. a) Schematic of the experimental setup for scattermetry. The narrowband radiation from the output port of a monochromator is impigning on the specimen at an angle of incidence $\theta_{in} = 70°$ while diffracted light beams at various scatter angles $(\theta_{scatter})$ are measured by rotating a photo-spectrometer. b) Reflectance vs. scattering angles of a palladium sample integrating an array of pixels with two structural colorations. The presence of two peaks for each incident wavelengths, namely $\lambda_{in} =$ 472 nm, 532 nm, and 633 nm, confirms existence of two superimposed diffraction gratings on the area probed by the incident beam.

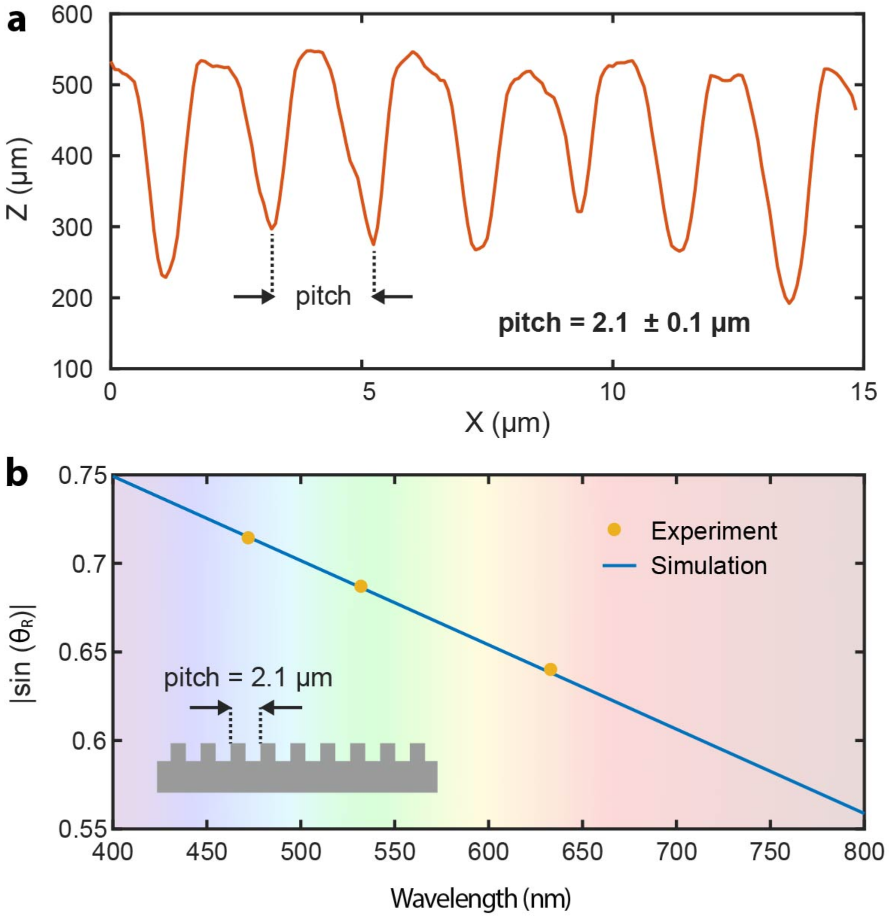

Figure S8. Reflective diffraction grating with AOF formed periodic structures on palladium. a) Cross-sectional profile of the structures obtained with an acoustic frequency of 1.2 MHz. The profile was extracted by the AFM map of Figure S6 along the x-axis. An average periodicity of 2.1 μm is evidently visible. b) Absolute value of the sinusoidal function evaluated at angles diffracted by an ideal reflective grating with a pitch of 2.1 μm (blue line) and measured on our sample (yellow symbol) with scatterometry. The perfect agreement between experiment and simulation is clearly evident.

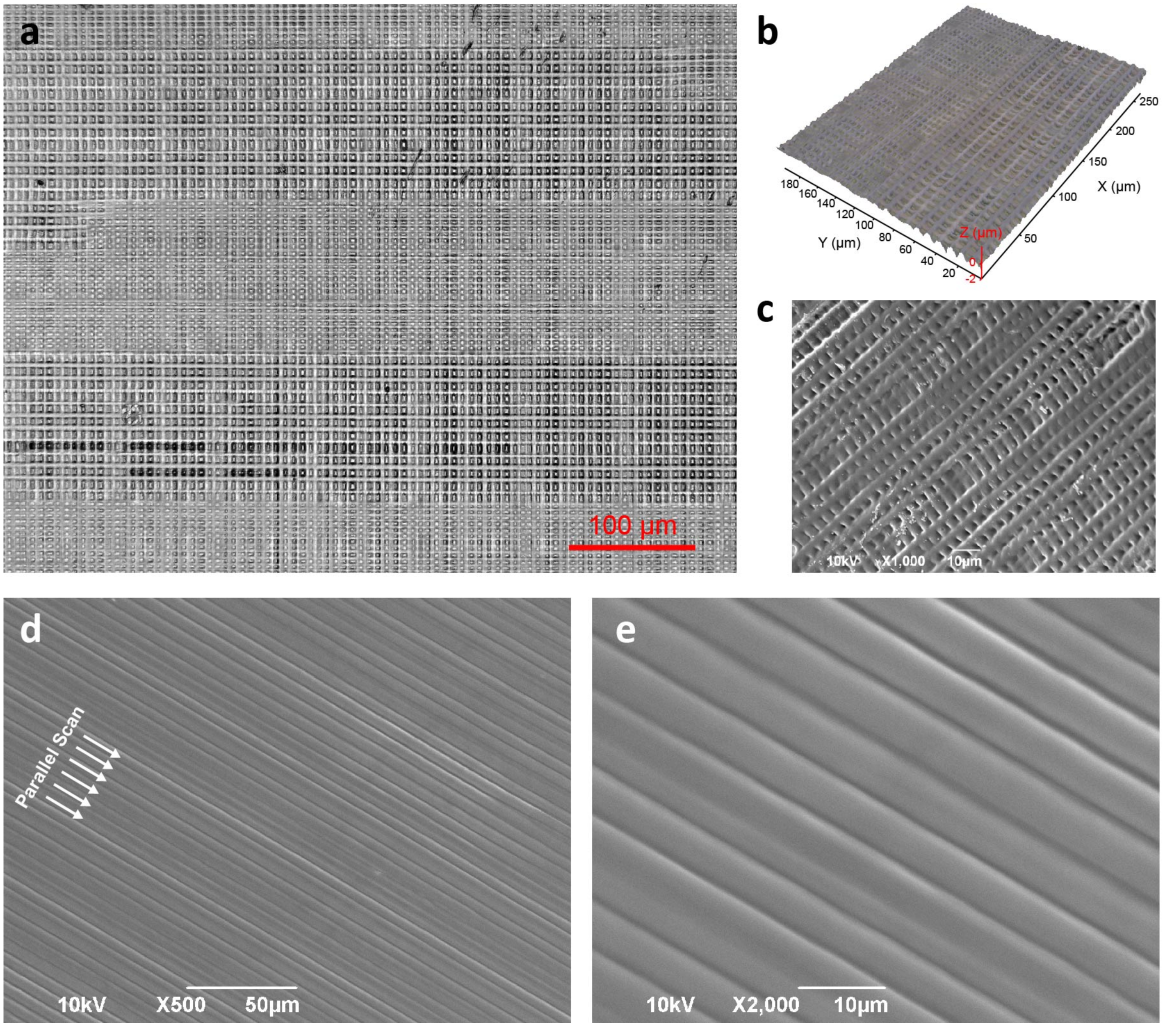

Figure S9.Large-area two-photons polymerization with acoustically shaped beams. a) Optical image and b) 3D optical profile of polymeric regular structures obtained by snake scanning an 800-nm pulsed laser at 0.3 mm/s. Scanning was performed twice, first along the x-axis with an acoustic frequency of 1.2 MHz and then along the y-axis while the acoustic frequency was binary modulated from 1.2 MHz to 1.8 MHz. c) Scanning electron micrograph (SEM) of the polymeric structures shown in (a). d) SEM of periodic polymeric lines obtained by snake scanning an 800-nm pulsed laser at 0.3 mm/s while the acoustic frequency in the scanning direction was 1.2 MHz. e) SEM at higher magnification highlights the high quality in term of surface roughness of the polymerized structures.

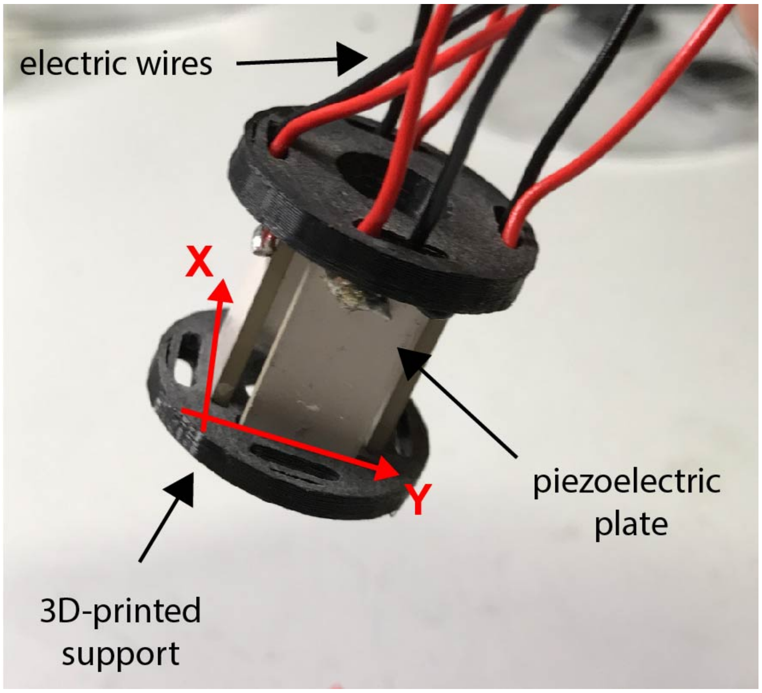

Figure S10. Assembly of the acusto-optofluidic system. Optical image of the core component of the AOF system consisting of two pairs of parallel piezoelectric plates aligned along two perpendicular directions (x and y in the figure). Two 3D-printed plastic elements allow simple assembling of the cavity while simultaneously ensuring each to be parallel and orthogonal between the plates. Electric wires soldered to both faces of each plate allow proper routing of driving signals.

Parameters	Value	Description
L	$1 \cdot 10^{-2}$	distance between piezoelectric plates, in meters
l	$2 \cdot 10^{-2}$	length of cavity, in meters
n_0	1.33	refractive index of water
c_s	1490	speed of sound in water, in m/s
μ	$1 \cdot 10^{-3*}$	dynamic shear viscosity of water at 20°C, in Pa.s
μ_b	$3 \cdot 10^{-3*}$	dynamic bulk viscosity of water at 20°C, in Pa.s
v_A	$1 \cdot 10^{-5}$	velocity of piezoelectric walls when driven at 10 V, in m/s
M	1	magnification of the 4f system
D	$1 \cdot 10^{-2}$	diameter of AOF system aperture, in m
ω_{input}	$2 \cdot 10^{-2}$	beam waist of incident Gaussian beam, in m

Table 1. Values for the base case parameters used in the simulations. *The viscosity values are extracted from Ref. [5].

References

1. McLeod, E. & Arnold, C.B. Mechanics and refractive power optimization of tunable acoustic gradient lenses, *J. Appl. Phys.* **102**, 033104 (2007)

2. Duocastella, M., & Arnold, C.B. Transient response in ultra high-speed liquid lenses, *J. Phys. D:Appl. Phys.* **46**, 075102(2013)

3. Goodman, J.W. Introduction to Fourier Optics, Third Edition (WH Freeman & Co, 2005)

4. Piazza, S., Bianchini, P., Sheppard, C., Diaspro, A. & Duocastella, M. Enhanced volumetric imaging in 2-photon microscopy via acoustic lens beam shaping. *J. Biophotonics* **11**, e201700050 (2018).

5. Xu J., Ren X., Gong W., Dai R. & Liu D Measurement of the Bulk Viscosity of Liquid by Brillouin Scattering, *Appl. Opt.* **42** 6704 (2003)
